# Data concerning the impact of mental fatigue on vigor as measured with the Brunel Mood Scale (BRUMS) in both physically active and trained subjects: A mini meta-analysis

**DOI:** 10.1016/j.dib.2017.06.056

**Published:** 2017-07-05

**Authors:** Maamer Slimani, Nicola Luigi Bragazzi

**Affiliations:** a Research Laboratory ‘‘Sport Performance Optimization’’, National Centre of Medicine and Science in Sport (CNMSS), El Menzah, Tunisia; b School of Public Health, Department of Health Sciences (DISSAL), Genoa University, Genoa, Italy; c Department of Neuroscience, Rehabilitation, Ophthalmology, Genetics, Maternal and Child Health (DINOGMI), Section of Psychiatry, Genoa University, Genoa, Italy

**Keywords:** Mental fatigue, Meta-regression, Mini meta-analysis, Vigor

## Abstract

The aim of the present mini meta-analysis was to compute the overall effect of mental fatigue induced by two cognitive tasks (namely, AX-continuous performance test or AX-CPT and incongruent Stroop test) on mood state, particularly on vigor, assessed using the Brunel Mood Scale (BRUMS), in both physically active and trained subjects. Search strategy was taken from a systematic review recently carried out by Van Cutsem and collaborators [1], as well as the quality assessment of the included studies. Mental fatigue was not found to impact on vigor score (*n*=62, effect size=0.12, −0.22–0.46, *p*=0.500, *Q*=0.18, *I*^2^=0.00, *T*^2^=0.00, *p*=0.996). However, an evidence of publication bias was found.

**Specifications Table**

**Table:** 

Subject area	*Sports sciences*
More specific subject area	*Sports Psychology*
Type of data	*Raw and analyzed*
How data was acquired	*Data were acquired from a systematic review recently carried out by Van Cutsem et al.**[1]**and from articles included in the current meta-analysis.*
Data format	*Table and figures*
Experimental factors	*Articles were included according to the inclusion/exclusion criteria stated in the systematic review by Van Cutsem et al.**[1]**. Quality assessment of the included studies was also taken from**[1]**. Data concerning publication year, gender, age, anthropometric features, training level, type and length of intervention, and study design were extracted from the included studies.*
Experimental features	*Meta-analysis (overall fixed-effect model) and meta-regressions according to different moderator variables (publication year, gender, age, training level, and type and length of intervention) were performed. Publication bias was also assessed.*
Data source location	*NA*
Data accessibility	*Data are within this article.*

**Value of the data**

• The effect of mental fatigue induced by cognitive tasks in both physically active and trained subjects was computed by performing a meta-analysis of pertinent studies.
• No significant difference was found between AX-continuous performance test or AX-CPT and incongruent Stroop test, in inducing mental fatigue.
• As an evidence of publication bias was found, further studies are urgently needed in the field.

## 1 Data

Mental fatigue is a psychobiological state that can be caused/induced by prolonged periods of demanding cognitive activity [1]. Previous studies have examined the effect of mental fatigue on physical performance, using the incongruent Stroop task or the AX-continuous performance test (AX-CPT) [2], [3], [4], [5], [6]. The aim of the present meta-analysis was to compute the effect of mental fatigue on mood state, particularly on vigor, and to determine the main moderator variables.

## 2 Experimental design, materials and methods

### 2.1 Design, materials and methods

Search strategy was taken from [1], as well as the quality assessment of the included studies.

All included studies used either incongruent Stroop task or AX-CPT as cognitive tasks to induce mental fatigue. The Brunel Mood Scale (BRUMS) developed by Terry et al. [7] was used to assess mood in all included studies. This questionnaire, which is based on the Profile of Mood States (POMS), contains 24 items (e.g., angry, uncertain, miserable, tired, nervous, energetic) divided into six respective subscales: anger, confusion, depression, fatigue, tension, and vigor. Of particular interest in the present meta-analysis was the subscale for vigor.

### 2.2 Meta-analysis

The meta-analysis was performed using the commercially available dedicated software Pro-meta (version 2.0, Internovi, Italy); alpha was set at *p*<0.05.

For the meta-analysis part, data were extracted from the included studies using a standardized documentation form. The parameters extracted included the surname of first author and year of publication, sample size, percentage of male and female, training level, type and length of intervention (cognitive task), and study design. Effect size (ES) was computed with its 95% confidence interval (CI). Additional analyses were performed after stratification by publication period, gender, training level, type and length of intervention.

Statistical heterogeneity was also assessed in our meta-analysis, using the *I*^2^ statistics. More in details, if the *I*^2^ was >50%, this was regarded as substantial heterogeneity. In case of significant heterogeneity, an overall random-effects model was preferred to an overall fixed-effect model. To identify sources of variation, further stratification was performed relative to study quality as assessed by [1]. In addition, for the sensitivity analyses, the stability of the pooled estimate with respect to each study was investigated by excluding individual studies from the analysis.

Possible publication bias was visually inspected with a funnel plot, looking at asymmetry of the graph. If asymmetry was present based on visual assessment, exploratory analyses were performed in order to investigate and adjust this using trim and/or fill analysis. In addition, the probability of publication bias was tested using Egger׳s linear regression, being significant of bias publication in case of *p*<0.10.

Sample size went from 10 to 16 subjects. Male percentage ranged from 62.5% to 100%. 22 subjects were considered physically active, whilst 40 individuals were considered trained. 38 subjects were exposed to AX-CPT, whilst 24 to the incongruent Stroop test. Studies were published from 2009 to 2015 [2], [3], [4], [5], [6] (Table 1).

**Table 1:** Effect of mental fatigue on vigor score according to the different studies included in the current meta-analysis.

**Study**	**Sample**	**Study design**	**Intervention**		**Control**		
			**Before**	**After**	**Before**	**After**	
*AX-CPT intervention vs. control (watching a documentary)*							
Marcora et al. [2]	*n*=16; healthy trained; 10 male and 6 female; age 26±3 y; height 175±9 cm, weight 69±10 kg, peak power output 288±70 W, peak oxygen uptake (V̇_O2peak_) 52±8 ml kg^−1^ min^−1^	Single-blind, randomized, counterbalanced, crossover experimental design; 90 min	6.2±3.1	3.5±2.2	6.2±3.1	3.5±2.2	
Pageaux et al. [5]	*n*=10; active; male; age 22±2 y; height: 177±6 cm, weight: 70±8 kg	Randomized, counterbalanced, crossover experimental design; 90 min	9.0±0.9	6.5±0.9	9.7±0.6	7.1±0.7	
Smith et al. [6]	*n*=10; healthy competitive intermittent team (soccer, Australian football, rugby league, rugby union, or field hockey) sportsmen; male; age 22±2 y; height 181±4 cm; weight 75±6 kg; V̇_O2max_ 48±6 ml kg^−1^ min^−1^	Randomized, counterbalanced, crossover experimental design; 90 min	7.6±2.7	3.3±2.5	7.6±2.7	3.3±2.5	

*100% incongruent modified Stroop color–word task vs. control (100% congruent Stroop color–word task)*							
Pageaux et al. [4]	*n*=12; healthy trained; 8 male and 4 female; age 21±1 y; height 174±12 cm, weight 69±11 kg	Randomized, counterbalanced, crossover experimental design; 30 min	5.9±1.1	4.2±1.0	6.1±1.4		4.6±1.5
Pageaux et al. [3]	*n*=12; healthy active; male; age 25±4 y; height: 182±5 cm, weight: 77±11 kg	Randomized, counterbalanced, crossover experimental design; 30 min	10.2±3.0	8.3±3.9	10.6±4.0		7.8±4.7

**Fig. 1:**
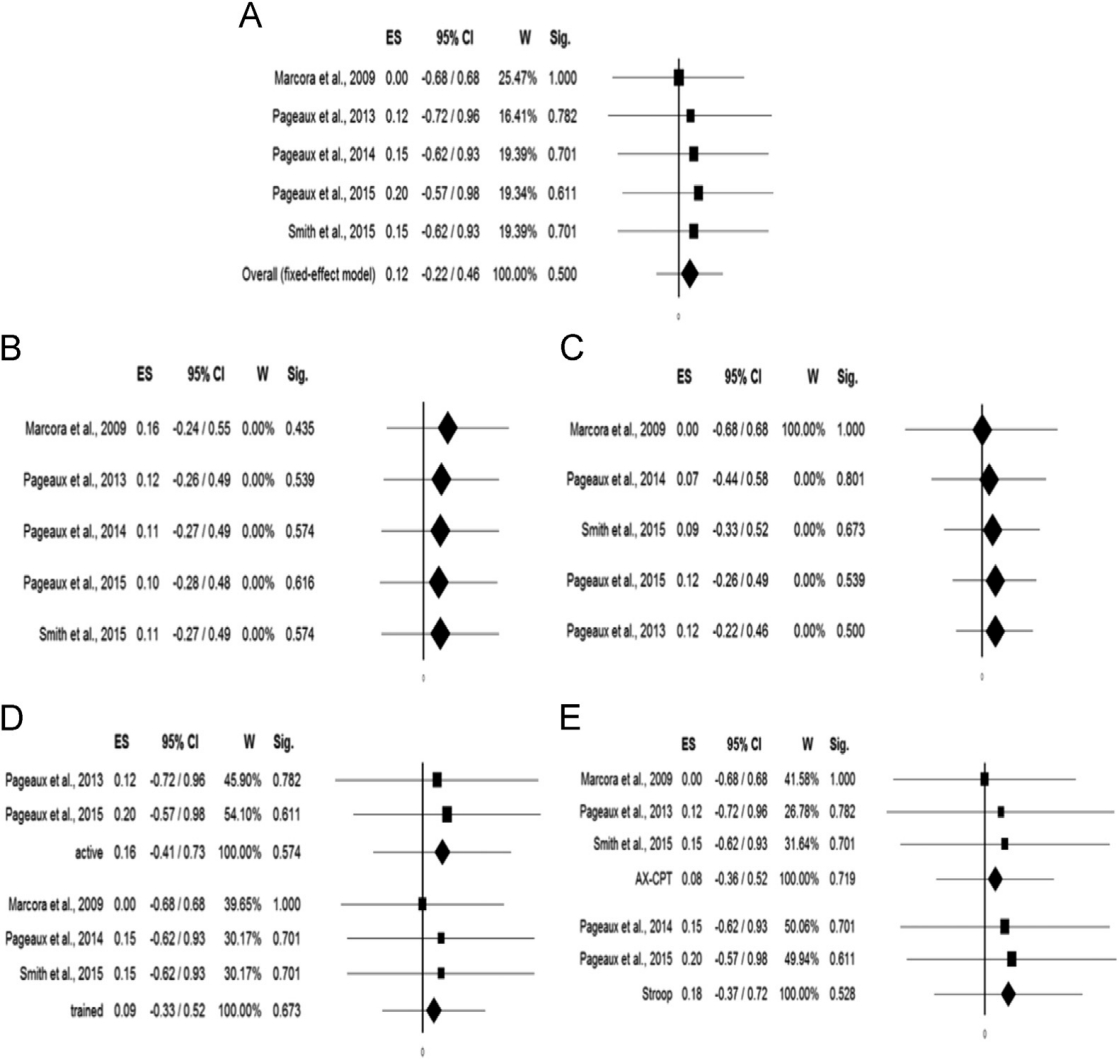
**A)** Meta-analysis of all studies, **B)** Sensitivity analysis, **C)** Cumulative meta-analysis, **D)** Meta-analysis stratified according to the training level of the studied subjects, **E)** Meta-analysis stratified according to the type of intervention.

**Fig. 2:**
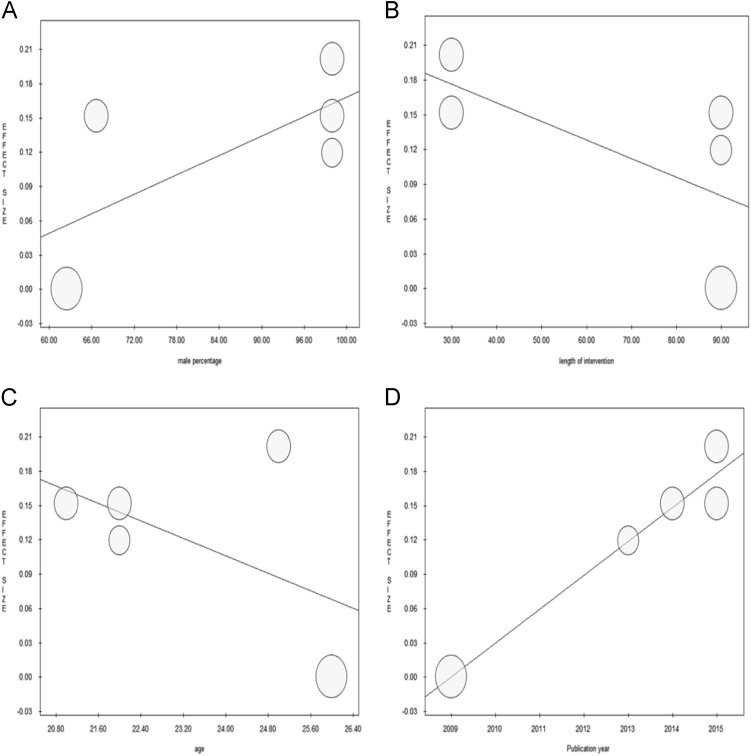
**A)** Meta-regression performed with male percentage as moderator, **B)** Meta-regression performed with length of the intervention as moderator, **C)** Meta-regression performed with age as moderator, **D)** Meta-regression performed using publication year as moderator.

Since heterogeneity was insignificant, an overall fixed-effect model was utilized. Mental fatigue was not found to impact on vigor score (*n*=62, ES=0.12, −0.22–0.46, *p*=0.500, *Q*=0.18, *I*^2^=0.00, *T*^2^=0.00, *p*=0.996) (Fig. 1A). Sensitivity and cumulative meta-analyses are shown in Fig. 1B and C, respectively. When stratifying according to the training level, no significant differences could be found between studies conducted with physically active or trained subjects (ANOVA Q-test for fixed-effect model, *Q*=0.04, *p*=0.843) (Fig. 1D). When stratifying according to the type of intervention, no differences could be detected (ANOVA Q-test for fixed-effect model, *Q*=0.07, *p*=0.787) (Fig. 1E). Performing meta-regressions, moderator variables such as male percentage (*p*=0.196), length of intervention (*p*=0.240), and age (*p*=0.361) were not found to be statistically significant (Fig. 2A–C).

**Fig. 3:**
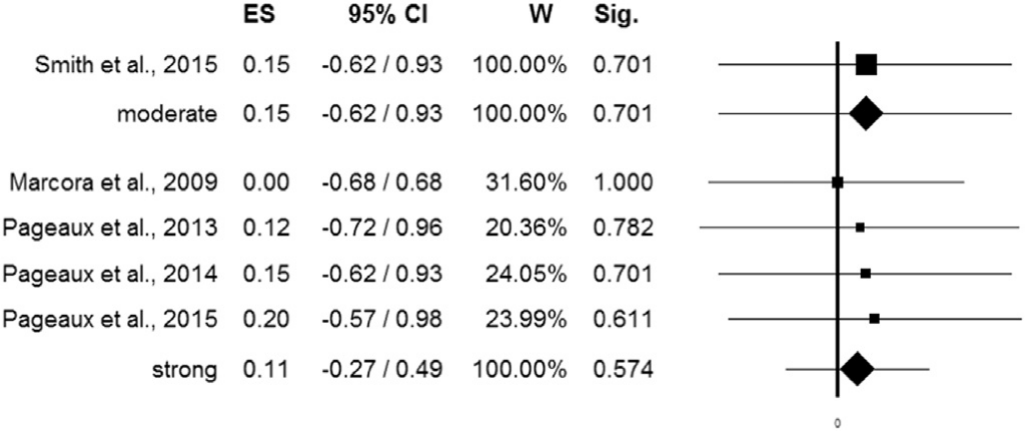
Meta-analysis stratified according to quality assessment.

When performing meta-regression with publication year as moderator variable, a significant trend was found (*p*=0.004) (Fig. 2D). Stratifying according to quality assessment, no statistical significance could be noticed (ANOVA Q-test for fixed-effect model, *Q*=0.01, *p*=0.923) (Fig. 3).

**Fig. 4:**
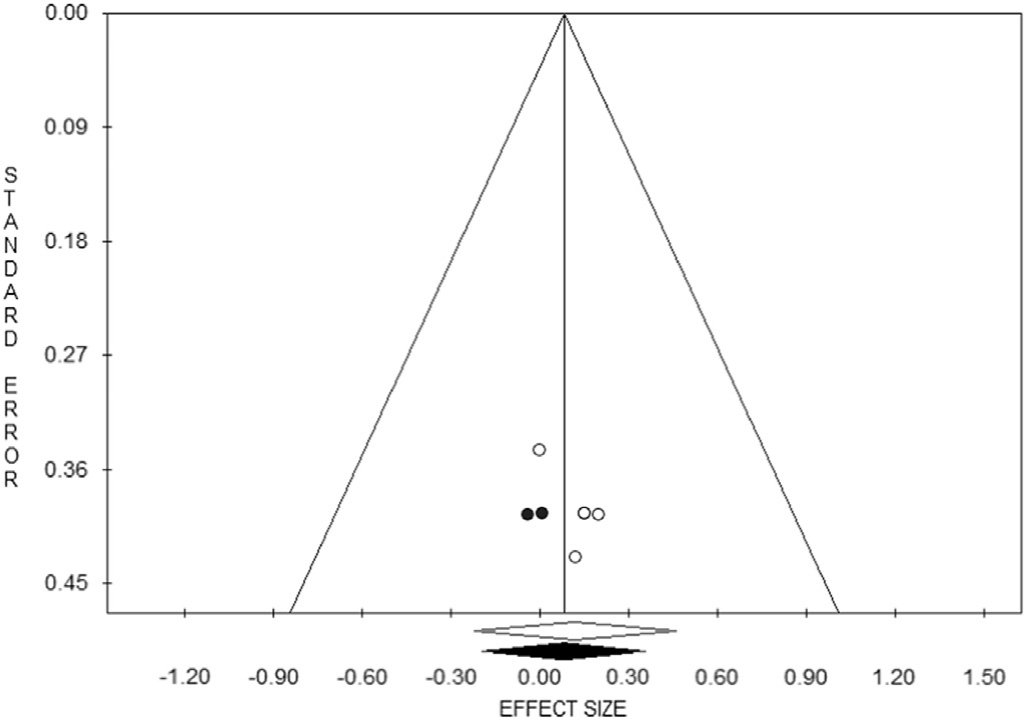
Publication bias as visually inspected with the funnel plot.

By visually inspecting the funnel plot and looking for asymmetry, evidence of publication bias could be found (Fig. 4), even though the Egger׳s linear test did not result statistically significant (intercept=1.93, *t*=1.92, *p*=0.151), as well as the Begg and Mazumdar׳s rank-correlation test (Z value for Kendall׳s tau=0.73, *p*=0.462). The trim and fill analysis showed, instead, two trimmed studies (estimated ES=0.08, −0.21–0.37, *p*=0.589).
